# Enhanced vitamin A production by engineering transporters, ATP and precursor supply

**DOI:** 10.1016/j.bidere.2025.100023

**Published:** 2025-04-17

**Authors:** Yijun Zhang, Qiongyue Hu, Hongwei Yu, Lidan Ye

**Affiliations:** a Key Laboratory of Biomass Chemical Engineering (Education Ministry), College of Chemical and Biological Engineering, Zhejiang University, Hangzhou, 310058, China; b Institute of Bioengineering, College of Chemical and Biological Engineering, Zhejiang University, Hangzhou, 310058, China

**Keywords:** Vitamin A, ABC transporters, ATP supply, *Saccharomyces cerevisiae*, Metabolic engineering

## Abstract

Vitamin A (retinoids) is essential for human metabolism and has extensive applications in medicine, health, and cosmetics. Microbial cell factories have been developed for retinoid biosynthesis, coupled with two-phase fermentation for *in situ* extraction. Given that notable portions of retinoids remained in the cells, promotion of retinoid secretion is expected for further production improvements. This study investigates the potential of yeast endogenous PDR family proteins to enhance retinoid efflux by overexpressing them in retinoid-producing *Saccharomyces cerevisiae* strains. Among the PDR proteins tested, the transcriptional factor Pdr3p and the transporters Pdr10p and Snq2p significantly enhanced retinol and retinal secretion and production, while the transcriptional factor Pdr8p and the transporters Pdr11p, Pdr12p, Pdr18p, and Aus1p markedly increased retinoic acid production. *PDR3*/*PDR10* co-overexpression improved retinal production to a record 638.12 ​mg/L, while *PDR8* overexpression led to 106.75 ​mg/L retinoic acid production in shake flasks. For retinol, synergistic overexpression of *PDR3* and *PDR10* elevated the extracellular proportion to 96.7 ​%. Given the ATP requirement of PDR protein-mediated transportation, ATP supply was strengthened by overexpressing the mitochondrial fusion-related gene *MGM1* and introducing hemoglobin Vgb, both enhancing retinol secretion and production. Further precursor supply enhancement resulted in 727.30 ​mg/L retinol from 20 ​g/L glucose in shake flasks, with a carbon conversion rate of 7.62 ​%. These results confirm the combination of transport engineering, energy regulation and precursor supply enhancement as a pivotal strategy for augmenting vitamin A production.

## 1 Introduction

Vitamin A, also known as retinoids, is an essential fat-soluble vitamin that plays diverse roles in vision, bone development, immune function, skin health, and cancer risk reduction [1]. Particularly, as the main active form in vitamin A, retinol is essential for vision health and acts as an important ingredient in the cosmetics industry with excellent antioxidant and anti-aging effects. Currently, commercial vitamin A is primarily produced through chemical synthesis, which faces challenges such as complex reaction steps, low yields, harsh conditions, and high material costs [2].

An emerging alternative is the biosynthesis of vitamin A using biomass-derived sugars as cost-effective feedstocks. This process can be achieved by introducing β-carotene 15,15′-monooxygenase (BCMO) into β-carotene-producing organisms. Potent β-carotene producers have been developed by engineering microbes [[3], [4], [5]]. BCMO catalyzes the cleavage of β-carotene into two retinal molecules, which can then be converted to retinol or retinoic acid through subsequent reduction or oxidation reactions. Successful biosynthesis of vitamin A has been reported in metabolic engineering efforts involving *Escherichia coli* [[6], [7], [8], [9], [10]], *S. cerevisiae* [[11], [12], [13], [14], [15], [16], [17], [18]], and *Yarroawia lipolytica* [[19], [20], [21]], via strategies of screening or modifying rate-limiting enzymes, mevalonate pathway enhancement, and cofactor engineering. Notably, *S. cerevisiae* has been engineered for selective biosynthesis of retinol [12] and retinal [14], as well as efficient biosynthesis of retinoic acid [13]. However, current yields remain insufficient to meet industrial demands.

The intracellular accumulation of hydrophobic compounds like carotenoids and retinoids can impose a metabolic burden [22]. *In situ* extraction methods using biphasic fermentation have been found to enhance production by facilitating the removal of these products [11,12]. Despite this, not all retinoids are secreted into the organic phase; a considerable proportion remains intracellular. For instance, while the addition of dodecane improved the excretion of the retinal/retinol mixture produced from xylose, about 17 ​% of vitamin A was still detected within the cells [11]. Similarly, during biphasic fermentation of a yeast strain selectively producing retinol, approximately 20 ​% of retinol was found intracellularly [12]. In the case of retinoic acid production, biphasic fermentation using dodecane or olive oil as extractants resulted in lower yields compared to monophasic fermentation due to the loss of retinal as a key precursor to the organic phase [13]. Therefore, identifying specific transporters for vitamin A compounds is crucial for enhancing the selective secretion of target products and overcoming existing production bottlenecks.

In recent years, transporter engineering has emerged as a promising strategy to enhance the secretion and production of terpenoids by improving cell tolerance and alleviating intracellular storage stress. The overexpression of ABC transporters has been shown to effectively improve bisabolene production in engineered *Y*. *lipolytica* [23] and boost astaxanthin production in engineered *Phaffia rhodozyma* [24]. The pleiotropic drug resistance (PDR) protein subfamily represents the largest subset within the ABC transporter superfamily and plays pivotal roles in various physiological processes, including sterol import, metal stress response, oxidative stress management, lipid transport, multidrug resistance, weak acid stress tolerance, and mating factor secretion. These functions enable cells to adapt to diverse environmental stresses [25]. Notably, the PDR regulators Pdr1p and Pdr3p, which control the expression of the PDR transporters, have been found to be upregulated in strains with high production of carotenoids [26] and artemisinic acid [27]. Their overexpression has also been linked to enhanced biosynthesis of carotenoids [28] and tocotrienols in *S. cerevisiae* [29]. Several studies have explored enhancing terpenoid secretion through the overexpression of various PDR transporters. For instance, Pdr10p has been identified as crucial for carotenoid secretion in both *S. cerevisiae* [30] and the oleaginous yeast *Rhodosporidium toruloides* [31]. Additionally, NtPDR1 from *Nicotiana tabacum* is involved in the transport of diterpenoids such as cembrene and sclareol [32]. Interestingly, PDR family proteins exhibit varying preferences for different terpenoids, even when these compounds share similar molecular structures. For example, Pdr10p was effective in transporting all forms of tocotrienols, while Pdr11p and Yol075cp demonstrated outstanding performance in secreting a tocotrienol mixture [29] but showed poor performance when secreting δ-tocotrienol as a single product [33]. Given these findings, it would be valuable to screen for PDR proteins capable of transporting retinoids and to investigate their preferences for the structurally similar retinol, retinal and retinoic acid.

As ABC proteins, the transport process mediated by PDR transporters requires a sufficient supply of ATP. For instance, the overexpression of *SNQ2* combined with increased ATP availability through acetate addition in *S. cerevisiae* resulted in a 5.80-fold increase in β-carotene secretion [30]. Additionally, overexpression of mitochondrial fusion-related genes *FZO1* and *MGM1* can further enhance ATP supply [34]. The introduction of hemoglobin Vgb, which improves O_2_ transport in the periplasm, has been shown to increase intracellular ATP and NADH/NAD^+^ ​levels in *Halomonas bluephagenesis* TD01, thereby enhancing the production of 3-hydroxybutyrate [35]. Combinatorial engineering aimed at boosting ATP supply through promoting mitochondrial fusion and improving oxygen availability may further enhance the secretion and production of retinoids.

In this study, we first overexpress the full range of endogenous PDR family proteins individually in previously constructed *S. cerevisiae* strains producing a mixture of retinal and retinol [12] or a mixture of retinal and retinoic acid [13]. This allows us to identify which PDR family proteins enhance retinoid efflux and synthesis. During this process, we also investigate and discuss the different preference of PDR transporters towards different vitamin A forms. Subsequently, we strengthen ATP supply by promoting mitochondrial fusion and improving oxygen supply together with overexpressing PDR proteins in combinations. After confirming a synergistic effect between transporter engineering and ATP enhancement, we apply this strategy to construct efficient yeast strains for selective production of retinol and retinal. This study not only establishes efficient yeast cell factories for secretory and selective production of three forms of vitamin A but also provides insights into understanding the preferences of PDR transporters for structurally similar compounds.

## 2 Materials and methods

### 2.1 Strains, culture media, reagents

*E. coli* strain BL21 was used for plasmid construction unless otherwise specified. The p423-ccdB plasmid was expressed in *E. coli* strain DB3.1. *S. cerevisiae* strains Y03 and Y03-252, both producing a mixture of retinal and retinol [12], Y03-43 selectively producing retinol [12], and Y03-784 primarily producing retinoic acid [13] were obtained from laboratory stock.

Luria-Bertani (LB) medium was used for the cultivation of *E. coli*, while Yeast Extract Peptone Dextrose (YPD) medium was utilized for the cultivation of *S. cerevisiae* strains. Synthetic Dropout (SD) medium omitting the corresponding amino acid(s) was used for selection of constructed *S. cerevisiae* strains. Ammonium sulfate was added as the nitrogen source to the SD medium for strain construction using the pUMRI plasmid. Sodium glutamate was added as the nitrogen source in the SD medium when utilizing CRISPR-Cas9 for strain construction. G418 was included in the SD medium for selecting engineered strains. Uracil and 5-fluoroorotic acid were added to the SD medium for the removal of the URA marker in engineered strains.

Tryptone was procured from Angel Yeast Co., Ltd. (Yichang, China), while yeast extract, peptone, and antibiotics were obtained from Sangon Biotech Co., Ltd. (Shanghai, China). YNB was sourced from Becton Dickinson Co., Ltd. (Shanghai, China). Standard compounds for retinol, retinal, and retinoic acid were obtained from Shanghai Yuanye Bio-Technology Co., Ltd. (Shanghai, China). High-fidelity DNA polymerase (DNA Prime STAR™ HS), DNA restriction endonucleases, and T4 DNA ligase were obtained from Takara Co., Ltd. (Dalian, China). High-fidelity DNA polymerase KOD-one was purchased from Toyobo Co., Ltd. (Shanghai, China). The 2 ​× ​Super PCR Mix DNA polymerase was obtained from Tsingke Co., Ltd. (Hangzhou, China), while DNA gel purification kits, cleanup kits, and plasmid extraction kits were purchased from Axygen Co., Ltd. (Hangzhou, China). Gene sequencing services were conducted by Tsingke Co., Ltd.

### 2.2 Construction of plasmids and strains

Strains for gene overexpression were constructed using the pUMRI plasmids [36]. Target genes were introduced into the multicloning sites of the plasmids through restriction digestion and ligation. Strains for gene knockout were constructed using CRISPR-Cas9 with P426-SpSgH for single-site knockout, and P426-ccdB for multi-site knockout [37]. The P426-ccdB plasmid was constructed from P426-SpSgH plasmid using Golden-gate assembly. All plasmids were transformed into *S. cerevisiae* strains using the LiAc/SS-DNA/PEG method [38]. The *VGB* gene was codon-optimized and synthesized by Sangon Biotech Co., Ltd. (Shanghai, China). All primers were synthesized by Generay Biotech Co., Ltd. (Shanghai, China). Detailed information for plasmids and primers is provided in Additional Tables S1 and S2, respectively. Description of *S. cerevisiae* strains is provided in Table 1.

**Table 1:** *S. cerevisiae* strains used and constructed in this study.

Strain name	Parent strain	Genotype/description	Source
Y03	Ycarot-02	*ΔHO*::T_*TPS1*_-*tHMG1*-P_*GAL7*_-P_*GAL2*_-*CrtYB*-T_*PGK1*_-T_*CYC1*_-*CrtI*-P_*GAL1*_-P_*GAL10*_-*CrtE03M*-T_*ADH1*_, *ΔGAL1-7*:: T_*ADH1*_-*CrtYB*-P_*GAL10*_-P_*GAL1*_-*CrtI*-T_*CYC1*_, *ΔGAL80*:: *LEU, ΔLPP1*:: P_*GAL1*_*-BLH-*T_*CYC1*_	[12]
Y03-252	Y03	*ΔMOT3*:: T_*ADH1*_-*tPOS5*-P_*GAL10*_-P_*GAL1*_-*CrtE03M*-T_*CYC1*_	[12]
Y03-43	Y03	*ΔMOT3*:: T_*ADH1*_-*tPOS5*-P_*GAL10*_-P_*GAL1*_-*ENV9*-T_*CYC1*_, *ΔDPP1*:: P_*GAL2*_-*CrtE03M*-T_*PGK1*_-P_*GAL7*_-*ybbO*-T_*TPS1*_	[12]
Y03-784	Y03	*ΔMOT3:: T*_*PGK1*_-*HFD1*-*P*_*GAL2*_-*P*_*GAL7*_-*HFD1*-*T*_*TPS1*_ *ΔDPP1*:: *T*_*PGK1*_-*HFD1*-*P*_*GAL2*_-*P*_*GAL7*_-*HFD1*-*T*_*TPS1*_	[13]
Yretinol-01	Y03-43	*ΔROX1*:: P_*GAL1*_-*PDR1*-T_*CYC1*_	This study
Yretinol-02	Y03-43	*ΔROX1*:: P_*GAL1*_-*PDR3*-T_*CYC1*_	This study
Yretinol-03	Y03-43	*ΔROX1*:: P_*GAL1*_-*PDR5*-T_*CYC1*_	This study
Yretinol-04	Y03-43	*ΔROX1*:: P_*GAL1*_-*PDR8*-T_*CYC1*_	This study
Yretinol-05	Y03-43	*ΔROX1*:: P_*GAL1*_-*PDR10*-T_*CYC1*_	This study
Yretinol-06	Y03-43	*ΔROX1*:: P_*GAL1*_-*PDR11*-T_*CYC1*_	This study
Yretinol-07	Y03-43	*ΔROX1*:: P_*GAL1*_-*PDR12*-T_*CYC1*_	This study
Yretinol-08	Y03-43	*ΔROX1*:: P_*GAL1*_-*PDR15*-T_*CYC1*_	This study
Yretinol-09	Y03-43	*ΔROX1*:: P_*GAL1*_-*PDR18*-T_*CYC1*_	This study
Yretinol-10	Y03-43	*ΔROX1*:: P_*GAL1*_-*AUS1*-T_*CYC1*_	This study
Yretinol-11	Y03-43	*ΔROX1*:: P_*GAL1*_-*SNQ2*-T_*CYC1*_	This study
Yretinol-12	Y03-43	*ΔROX1*:: P_*GAL1*_-*YOR1*-T_*CYC1*_	This study
Yretinol-13	Y03-43	*ΔROX1*:: P_*GAL1*_-*YOL075C*-T_*CYC1*_	This study
Yretinol-21	Y03-43	*ΔROX1*:: T_*CYC1*_-*PDR3*-P_*GAL1*_-P_*GAL10*_-*PDR10*-T_*ADH1*_	This study
Yretinol-22	Y03-43	*ΔROX1*:: T_*CYC1*_-*PDR3*-P_*GAL1*_-P_*GAL10*_-*SNQ2-*T_*ADH1*_	This study
Yretinol-23	Y03-43	*ΔROX1*:: T_*CYC1*_-*PDR10*-P_*GAL1*_-P_*GAL10*_-*PDR3*-T_*ADH1*_	This study
Yretinol-24	Y03-43	*ΔROX1*:: T_*CYC1*_-*PDR10*-P_*GAL1*_-P_*GAL10*_-*SNQ2*-T_*ADH1*_	This study
Yretinol-25	Y03-43	*ΔROX1*:: T_*CYC1*_-*SNQ2*-P_*GAL1*_-P_*GAL10*_-*PDR3*-T_*ADH1*_	This study
Yretinol-26	Y03-43	*ΔROX1*:: T_*CYC1*_-*SNQ2*-P_*GAL1*_-P_*GAL10*_-*PDR10*-T_*ADH1*_	This study
Yretinol-31	Y03-43	*ΔROX1*:: T_*CYC1*_-*PDR3*-P_*GAL1*_-P_*GAL10*_-*FZO1*-T_*ADH1*_	This study
Yretinol-32	Y03-43	*ΔROX1*:: T_*CYC1*_-*PDR3*-P_*GAL1*_-P_*GAL10*_-*MGM1-*T_*ADH1*_	This study
Yretinol-33	Y03-43	*ΔROX1*:: T_*CYC1*_-*PDR10*-P_*GAL1*_-P_*GAL10*_-*FZO1*-T_*ADH1*_	This study
Yretinol-34	Y03-43	*ΔROX1*:: T_*CYC1*_-*PDR10*-P_*GAL1*_-P_*GAL10*_-*MGM1*-T_*ADH1*_	This study
Yretinol-35	Y03-43	*ΔROX1*:: T_*CYC1*_-*SNQ2*-P_*GAL1*_-P_*GAL10*_-*FZO1*-T_*ADH1*_	This study
Yretinol-36	Y03-43	*ΔROX1*:: T_*CYC1*_-*SNQ2*-P_*GAL1*_-P_*GAL10*_-*MGM1*-T_*ADH1*_	This study
Yretinol-37	Y03-43	*ΔROX1*:: P_*GAL1*_-*VGB*-T_*CYC1*_	This study
Yretinol-41	Yretinol-21	*Δ911B*	This study
Yretinol-42	Yretinol-21	*Δ911B*:: P_*GAL1*_-*VGB*-T_*CYC1*_	This study
Yretinol-43	Yretinol-21	*Δ911B*:: P_*GAL10*_-*MGM1*-T_*ADH1*_	This study
Yretinol-44	Yretinol-21	*Δ911B*:: T_*CYC1*_-*VGB*-P_*GAL1*_-P_*GAL10*_-*MGM1-*T_*ADH1*_	This study
Yretinol-51	Yretinol-44	*Int7*:: P_*GAL1*_-*ACS1*-T_*CYC1*_*, Int18*:: P_*GAL1*_-*ACS2*-T_*CYC1*_	This study
Yretinol-52	Yretinol-44	*Int7*:: P_*GAL1*_-*ACS1*-T_*CYC1*_*, Int18*:: P_*GAL1*_-*ACS*^*L641P*^-T_*CYC1*_	This study
Yretinol-53	Yretinol-51	*ΔMLS1*:: P_*GAL1*_-*ERG10*-T_*CYC1*_	This study
Yretinol-54	Yretinol-52	*ΔMLS1*:: P_*GAL1*_-*ERG10*-T_*CYC1*_	This study
Yretinal-01	Y03	*ΔROX1*:: P_*GAL1*_-*PDR1*-T_*CYC1*_	This study
Yretinal-02	Y03	*ΔROX1*:: P_*GAL1*_-*PDR3*-T_*CYC1*_	This study
Yretinal-03	Y03	*ΔROX1*:: P_*GAL1*_-*PDR5*-T_*CYC1*_	This study
Yretinal-04	Y03	*ΔROX1*:: P_*GAL1*_-*PDR8*-T_*CYC1*_	This study
Yretinal-05	Y03	*ΔROX1*:: P_*GAL1*_-*PDR10*-T_*CYC1*_	This study
Yretinal-06	Y03	*ΔROX1*:: P_*GAL1*_-*PDR11*-T_*CYC1*_	This study
Yretinal-07	Y03	*ΔROX1*:: P_*GAL1*_-*PDR12*-T_*CYC1*_	This study
Yretinal-08	Y03	*ΔROX1*:: P_*GAL1*_-*PDR15*-T_*CYC1*_	This study
Yretinal-09	Y03	*ΔROX1*:: P_*GAL1*_-*PDR18*-T_*CYC1*_	This study
Yretinal-10	Y03	*ΔROX1*:: P_*GAL1*_-*AUS1*-T_*CYC1*_	This study
Yretinal-11	Y03	*ΔROX1*:: P_*GAL1*_-*SNQ2*-T_*CYC1*_	This study
Yretinal-12	Y03	*ΔROX1*:: P_*GAL1*_-*YOR1*-T_*CYC1*_	This study
Yretinal-13	Y03	*ΔROX1*:: P_*GAL1*_-*YOL075C*-T_*CYC1*_	This study
Yretinal-21	Y03-252	*ΔADH6*, *ΔADH7*, *ΔGRE2*, *ΔSFA1*	This study
Yretinal-22	Y03-252	*ΔADH6*, *ΔADH7*, *ΔGRE2*, *ΔSFA1*, *ΔENV9*	This study
Yretinal-31	Yretinal-21	*ΔROX1*:: P_*GAL1*_-*PDR3*-T_*CYC1*_	This study
Yretinal-32	Yretinal-21	*ΔROX1*:: P_*GAL1*_-*PDR10*-T_*CYC1*_	This study
Yretinal-33	Yretinal-21	*ΔROX1*:: T_*CYC1*_-*PDR3*-P_*GAL1*_-P_*GAL10*_-*PDR10*-T_*ADH1*_	This study
Yretinal-34	Yretinal-21	*ΔROX1*:: T_*CYC1*_-*PDR10*-P_*GAL1*_-P_*GAL10*_-*PDR3*-T_*ADH1*_	This study
Yretinal-35	Yretinal-22	*ΔROX1*:: P_*GAL1*_-*PDR3*-T_*CYC1*_	This study
Yretinal-36	Yretinal-22	*ΔROX1*:: P_*GAL1*_-*PDR10*-T_*CYC1*_	This study
Yretinal-37	Yretinal-22	*ΔROX1*:: T_*CYC1*_-*PDR3*-P_*GAL1*_-P_*GAL10*_-*PDR10*-T_*ADH1*_	This study
Yretinal-38	Yretinal-22	*ΔROX1*:: T_*CYC1*_-*PDR10*-P_*GAL1*_-P_*GAL10*_-*PDR3*-T_*ADH1*_	This study
Yra-01	Y03-784	*ΔROX1*:: P_*GAL1*_-*PDR1*-T_*CYC1*_	This study
Yra-02	Y03-784	*ΔROX1*:: P_*GAL1*_-*PDR3*-T_*CYC1*_	This study
Yra-03	Y03-784	*ΔROX1*:: P_*GAL1*_-*PDR5*-T_*CYC1*_	This study
Yra-04	Y03-784	*ΔROX1*:: P_*GAL1*_-*PDR8*-T_*CYC1*_	This study
Yra-05	Y03-784	*ΔROX1*:: P_*GAL1*_-*PDR10*-T_*CYC1*_	This study
Yra-06	Y03-784	*ΔROX1*:: P_*GAL1*_-*PDR11*-T_*CYC1*_	This study
Yra-07	Y03-784	*ΔROX1*:: P_*GAL1*_-*PDR12*-T_*CYC1*_	This study
Yra-08	Y03-784	*ΔROX1*:: P_*GAL1*_-*PDR15*-T_*CYC1*_	This study
Yra-09	Y03-784	*ΔROX1*:: P_*GAL1*_-*PDR18*-T_*CYC1*_	This study
Yra-10	Y03-784	*ΔROX1*:: P_*GAL1*_-*AUS1*-T_*CYC1*_	This study
Yra-11	Y03-784	*ΔROX1*:: P_*GAL1*_-*SNQ2*-T_*CYC1*_	This study
Yra-12	Y03-784	*ΔROX1*:: P_*GAL1*_-*YOR1*-T_*CYC1*_	This study
Yra-13	Y03-784	*ΔROX1*:: P_*GAL1*_-*YOL075C*-T_*CYC1*_	This study

### 2.3 Culture and fermentation conditions

Single colonies were picked from YPD plate and incubated in a test tube with 5 ​mL YPD at 30 ​°C with shaking at 220 ​rpm overnight. The seed culture was then inoculated into a 250 ​mL shake flask containing 50 ​mL YPD for light-shielded cultivation over a period of 84 ​h. For strains undergoing two-phase fermentation, 5 ​% (v/v) dodecane was added as the organic phase, along with 1 ​% (w/v) butylated hydroxytoluene (BHT), an antioxidant used to prevent oxidation of secreted retinoids, and 1.44 ​mM Fe^2+^ was added to ensure BCMO activity. For strains producing retinoic acid, since extracting retinoic acid using organic solvent proved challenging and its precursor retinal could be extracted more easily, monophasic fermentation was conducted.

### 2.4 Analysis of carotenoids and retinoids

Carotenoids were analyzed according to the method used in previous study [36]. To determine extracellular retinoids in two-phase fermentation, 50 ​μL of organic phase samples were diluted with 950 ​μL acetone, and filtered through a 0.22 ​μm organic filter prior to HPLC analysis. To determine extracellular retinoids in single-phase fermentation, 1 ​mL aqueous phase samples were extracted with equal volume of ethyl acetate, followed by drying in a rotary evaporator and adding 1 ​mL of acetone before filtration and HPLC analysis. To determine intracellular retinoids, the cell pellet collected from 1 ​mL culture was resuspended in an equal volume of acetone, and the cells were disrupted at 65 ​Hz using grinding beads, followed by filtration and HPLC analysis. Meanwhile, the cell pellet collected from 5 ​mL of culture was dried in an oven at 100 ​°C till constant weight to measure the dry cell weight.

Retinoid quantification was performed on an HPLC system (SHIMADZU SPD-20A, Japan) equipped with a C18-H chromatographic column (4.6 ​× ​250 ​mm, 5 ​μm, YMC-Pack ODS-AQ), and the column temperature was set at 40 ​°C. The mobile phase consisted of a 2 ​% acetic acid solution (v/v) and acetonitrile, with a ratio of 7.5 ​%:92.5 ​%, and a flow rate of 0.6 ​mL/min^13^. HPLC detection was performed at a wavelength of 352 ​nm. For preparation of standard curves, the standard compounds were dissolved in methanol and subsequently diluted with acetone to different concentrations.

### 2.5 Molecular docking

The crystal structure of the PDR transporter Pdr5p utilized for molecular docking was obtained from the PDB database (https://www.rcsb.org/) [39,40], while the rest were predicted structures from the AlphaFold protein structure database (https://alphafold.ebi.ac.uk/) [41]. Molecular structures of retinal, retinol, and retinoic acid were retrieved from the ZINC database (https://zinc.docking.org/) [42]. Molecular docking was performed using AutoDockTools 1.5.7. After preprocessing the protein and ligands, the Genetic Algorithm was employed as the search parameter. The search algorithm used Genetic Algorithm (4.2), with the result showing the lowest binding free energy between the receptor protein and ligand considered as the optimal binding conformation. The molecular docking results were visualized using ChimeraX 1.7.

## 3 Results and discussion

### 3.1 Screening of PDR family proteins for promoting vitamin A secretion and production

To screen for transporters that facilitate vitamin A efflux and investigate their preference among different forms of vitamin A, we individually overexpressed all endogenous PDR family proteins, including three transcription factors (Pdr1p, Pdr3p, Pdr8p) and ten transporters, in previously constructed *S. cerevisiae* strain Y03 ^12^, which produces retinol and retinal, and Y03-784^13^ which produces retinal and retinoic acid. By observing changes in the titers and intracellular-to-extracellular ratios of different retinoids before and after overexpression of these proteins, we aimed to gain insights into the substrate preferences of different PDR family proteins. Considering that excessive expression of membrane proteins may impose a cellular burden, all PDR family proteins were overexpressed using P_*GAL1*_ in the background of *GAL80* knockout [43] to initiate their expression upon glucose depletion so as to separate their expression from cell growth in the same way as the vitamin A synthetic genes.

**Fig. 1:**
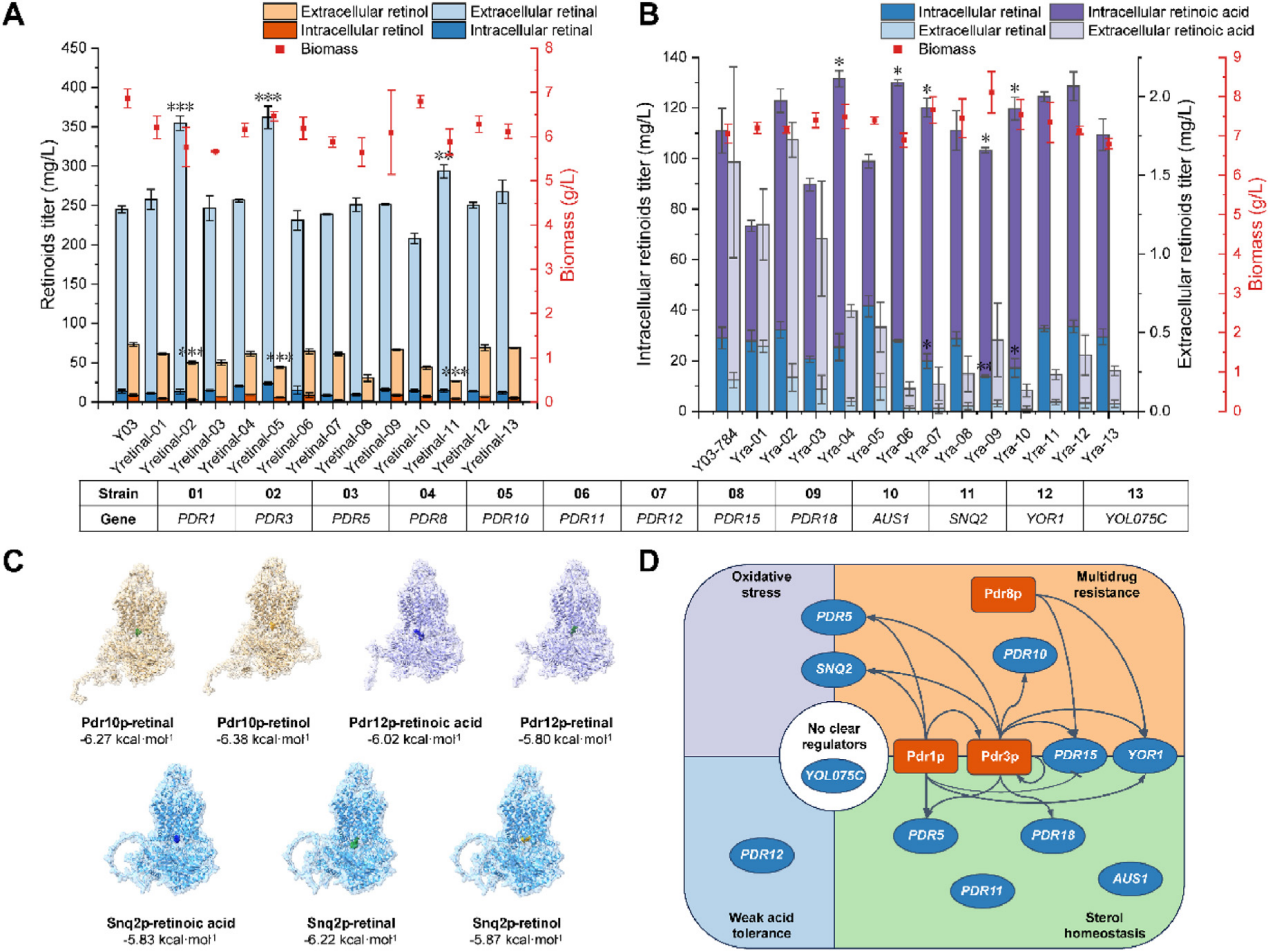
Effect of PDR family proteins on vitamin A production in *S. cerevisiae*. (A) Effect of overexpressing different PDR family proteins on retinol and retinal production in Y03. (B) Effect of overexpressing different PDR family proteins on retinoic acid and retinal production in Y03-784. (C) Molecular docking simulation of PDR transporters binding to retinoids. (D) Gene networks of *S. cerevisiae* PDR transcription factors (yellow) and transporters (blue). All values presented are the means of three biological replicates, and error bars represent standard deviations. Statistical analysis was performed using Student's t-test, ∗*P* ​< ​0.05, ∗∗*P* ​< ​0.01, ∗∗∗*P<* 0.001.

The overexpression of *PDR3*, *PDR10* and *SNQ2* in strain Y03 resulted in significant increases in retinoid titers (Fig. 1A). Compared to the control strain, the total retinoid titers increased from 317.89 ​mg/L to 404.59 ​mg/L, 406.36 ​mg/L and 320.06 ​mg/L, with the extracellular retinoid proportions changing from 92.9 ​% to 95.9 ​%, 92.6 ​% and 94.0 ​%, respectively. Specifically, the retinal production increased by 44.7 ​%, 47.8 ​% and 19.9 ​%, while the retinol titer decreased by 31.3 ​%, 39.1 ​%, and 63.5 ​%, respectively. Since retinal is the direct precursor for retinol, these results implied that retinal was exported more readily by these transporters than retinol. Pdr10p and Snq2p are crucial drug efflux pumps in *S. cerevisiae* responsible for multidrug resistance [44], and are also shown effective in transporting β-carotene [30], which is the direct precursor for retinoids. As hydrophobic diterpenes, retinoids may also be recognized as cargos of these transporters. According to molecular docking results, Snq2p exhibited significantly lower binding energy for retinal compared to retinol, while Pdr10p, showed a slightly lower binding energy for retinol (Fig. 1C), which may explain the more significant decrease in retinol titer in the Snq2p-overexpressing strain. The elevation effect of transcription factor Pdr3p likely arises from its regulation of Pdr10p and Snq2p, resulting in effects similar to those observed with Pdr10p and Snq2p. Furthermore, Pdr3p contains the UAS partly homologous to the *GAL* promoter, and its overexpression can enhance the expression of P_*GAL*_-driven genes [45]. This may be another reason why Pdr3p increased the retinal titer.

In strain Y03-784, overexpression of *PDR8*, *PDR11*, *PDR12*, *PDR18*, and *AUS1* significantly increased retinoic acid titer, while overexpression of *PDR3*, *SNQ2* and *YOR1* led to slight production improvement (Fig. 1B). Notably, *PDR8* overexpression led to the most significant increase in total retinoic acid, from 83.34 ​mg/L to 106.75 ​mg/L, a 28.1 ​% improvement compared to the control strain. Surprisingly, although some strains showed an increase in intracellular retinoic acid titers, overexpression of these transporters did not lead to an increase in extracellular retinoic acid titer. This result implied that the mechanism of PDR protein overexpression for improving retinoic acid production may not be simply increasing the product efflux. The eight aforementioned PDR family proteins can be classified into four categories: transcription factors (Pdr3p, Pdr8p), multidrug resistance-related transporters (Snq2p, Yor1p), weak acid tolerance-related transporter (Pdr12p), and sterol homeostasis-related transporters (Pdr11p, Pdr18p, Aus1p) (Fig. 1D). Both Pdr3p and Pdr8p have been reported to contribute to the toxic product tolerance of the strain by regulating the efflux pumps Snq2p and Yor1p [46]. Snq2p appears to have a transport function for all retinoids, with the highest selectivity for retinal, which was supported by the molecular docking results, where Snq2p showed the lowest binding energy with retinal (Fig. 1C). In addition, Yor1p might facilitate the transport of retinoic acid by participating in the flip-flop of lipids from the inner to outer membrane [47]. Additionally, Pdr3p may also enhance retinoic acid production by regulating Pdr18p, a transporter associated with the sterol composition of the plasma membrane [48,49]. The production increased by the overexpression of *PDR11* and *AUS1* may be linked to sterol import [[50], [51], [52]]. The elevations induced by Pdr11p, Pdr18p, and Aus1p may all result in restoration of ergosterol homeostasis, which assists cells in resisting weak acid-induced changes in nonspecific membrane permeability and transmembrane electrochemical potential [49]. Pdr12p, identified as a crucial transporter for *S. cerevisiae* in response to weak acid stress, possesses a natural response mechanism to retinoic acid-induced weak acid stress [53,54]. Molecular docking results also indicated that Pdr12p had the lowest binding energy when interacting with retinoic acid, consistent with its reported role as the primary weak acid efflux pump [53,54].

### 3.2 Transporter engineering to enhance selective production of retinol and retinal

**Fig. 2:**
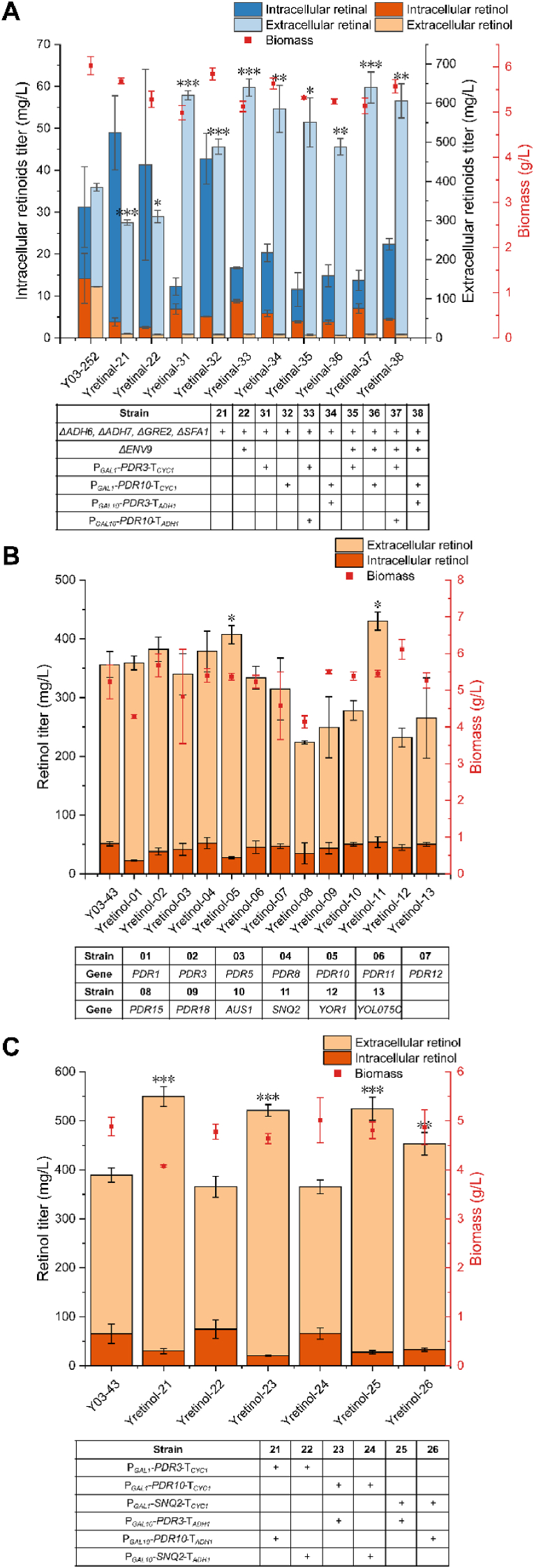
Effect of PDR family proteins on selective production of retinal and retinol. (A) Effect of dehydrogenases knockout and combinatorial transporter engineering on retinal production and biomass. (B) Effect of overexpressing different PDR family proteins on retinol production and biomass. (C) Effect of combinatorial transporter engineering on retinol production and biomass. All values presented are the means of three biological replicates, and error bars represent standard deviations. Statistical analysis was performed using Student's t-test, ∗*P* ​< ​0.05, ∗∗*P* ​< ​0.01, ∗∗∗*P<* 0.001.

To investigate the substrate specificity of PDR family proteins for retinal, we constructed an *S. cerevisiae* strain that selectively produces retinal. We aimed to reduce the retinol ratio in Y03-252 by knocking out the genes encoding four endogenous dehydrogenases: Adh6, Adh7, Gre2 and Sfa1 [14], generating strain Yretinal-21. Following the knockout of these dehydrogenases, the retinol proportion dropped from 34.9 ​% to 4.2 ​% (Fig. 2A). Further gene knockout of the previously identified endogenous dehydrogenase Env9 with retinal reduction activity [12] generated strain Yretinal-22, with a retinol proportion further decreased to 3.2 ​% (Fig. 2A). Although the total retinoid titers decreased in both knockout strains Yretinal-21 and Yretinal-22, the retinal titers increased from 270.93 ​mg/L to 328.99 ​mg/L and 340.58 ​mg/L, rising by 21.4 ​% and 25.7 ​%, respectively. Similar to Y03, overexpression of Pdr3p and Pdr10p individually or in combinations all exhibited significant promotion effects on retinal production in Yretinal-21 and Yretinal-22. The best-performing strain, Yretinal-33, was constructed by overexpressing *PDR3* under *GAL1* promoter and *PDR10* under *GAL10* promoter in Yretinal-21. It produced 638.12 ​mg/L of retinal, reflecting a 94.0 ​% improvement compared to the Yretinal-21 without transporter engineering. Meanwhile, the extracellular proportion of retinal in Yretinal-33 increased from 88.5 ​% to 98.7 ​%. The simultaneous improvement in retinal production and secretion suggests that overexpression of these PDR proteins does not only promote the efflux of the products but also drives the synthesis of the product.

In these efforts, PDR proteins effectively enhancing retinal secretion and synthesis were identified, however, their effects on retinol production were challenging to assess in strain Y03 producing a mixture of retinal and retinol due to the interference caused by export of retinal as the direct precursor for retinol. To identify PDR proteins that promote retinol production, we individually overexpressed all endogenous PDR family proteins in another *S. cerevisiae* strain, Y03-43 ^12^, which selectively produces retinol. Similar to retinal, Pdr3p, Pdr10p, and Snq2p were found to enhance retinol efflux and increase retinol titer in biphasic fermentation using dodecane as the organic phase (Fig. 2B). Unlike retinal, Snq2p outperformed other transporters, augmenting total retinol titer from 356.21 ​mg/L to 430.14 ​mg/L, a 20.8 ​% improvement, followed by Pdr10p which enhanced the retinol titer by 14.3 ​%. Moreover, overexpression of these transporters increased the extracellular retinol ratio from 85.6 ​% to 93.2 ​% and 87.4 ​%, respectively. Although Pdr10p has weaker transport capability compared to Snq2p, the higher proportion of extracellular retinol indicated that its selectivity was stronger (Fig. 2B).

The combinatorial overexpression of Pdr3p, Pdr10p, and Snq2p is expected to further enhance the transport efficiency of retinol. However, since PDR family proteins are membrane proteins, changes in expression intensity may have a significant impact. To address this issue, we employed *GAL* promoters with varying strengths, namely P_*GAL1*_ and P_*GAL10*_ [55], to overexpress these proteins. The results indicated that most combinatorial overexpression strains significantly increased the retinol production. Among them, Yretinol-21, which simultaneously overexpressed Pdr3p under P_*GAL1*_ and Pdr10p under P_*GAL10*_, outperformed the others, with a 41.2 ​% improvement in retinol titer (from 389.25 ​mg/L to 549.79 ​mg/L). Concurrently, the extracellular proportion of retinol increased from 83.2 ​% to 94.5 ​%. Strains Yretinol-22 and Yretinol-24 overexpressing *SNQ2* under the weaker P_*GAL10*_ and *PDR3 or PDR10* under the stronger P_*GAL1*_ did not show any increase in retinol titers, while Yretinol-25 and Yretinol-26 expressing *SNQ2* under P_*GAL1*_ and *PDR3 or PDR10* under P_*GAL10*_ showed significantly higher retinol production than the control strain. Similarly, the retinol titers in strains Yretinol-21 and Yretinol-23 were improved to different extents (by 41.24 ​%–34.01 ​%) when *PDR3* and *PDR10* were expressed under different promoters. These results underscore the importance of selecting appropriate promoter strength for overexpressing the transporters. Additionally, the increase in retinol titer (by 16.41 ​% vs. 41.2 ​%) and secretion proportion (by 29.71 ​% vs. 60.39 ​%) for Yretinol-26 overexpressing both Snq2p and Pdr10p was lower compared to Yretinol-21 overexpressing Pdr10p together with the transcriptional factor Pdr3p. This may be attributed to intensified ATP consumption resulting from excessive expression of transport proteins, thereby reducing the transport efficiency. In the transporter-overexpressing strains with higher retinol production, the color of the dodecane phase was generally more reddish (Fig. S1) due to the partial secretion of carotenoids by these transporters. This was further confirmed by HPLC analysis of the precursors in the organic phase (Fig. S2). This is in accordance with the previous studies where both Pdr10p and Snq2p were found to aid in secreting β-carotene [30].

### 3.3 Enhancement of mitochondrial fusion and oxygen supply to ensure ATP supply

Sufficient intracellular ATP levels are essential for ensuring the transport efficiency of ABC transporters. When strains overexpressing the ABC transporters experience inadequate ATP supply, their synthetic capabilities are diminished due to disrupted cellular metabolism [30]. In aerobic fermentation, ATP generation primarily relies on oxidative phosphorylation within the mitochondria. The process of mitochondrial fusion in *S. cerevisiae* involves the coordinated action of Ugo1p, Fzo1p, and Mgm1p [56]. The outer membrane protein Fzo1p collaborates with Mgm1p, which is located in the intermembrane space. These three proteins form a functional complex that drives mitochondrial fusion. Overexpressing *FZO1* or *MGM1* can facilitate mitochondrial fusion, preserving mitochondrial DNA integrity and consequently elevating ATP supply [34]. Therefore, *FZO1* and *MGM1* were overexpressed in strains that also overexpressed *PDR3*, *PDR10*, and *SNQ2*, which showed positive effects on retinol production.

**Fig. 3:**
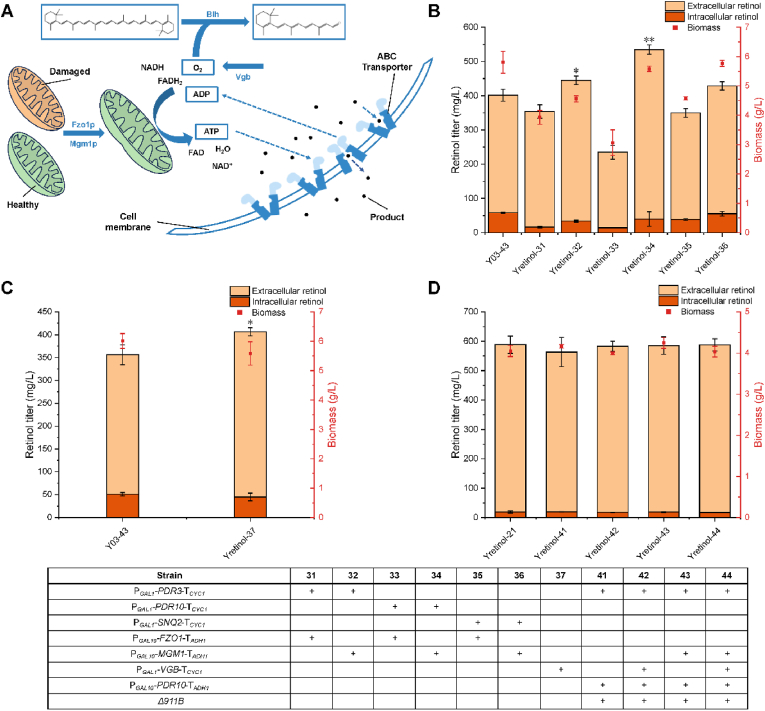
Effect of energy supply engineering on retinol production. (A) Strategies of ATP supply engineering for promoting retinol production. (B) Effect of overexpressing mitochondrial fusion-related genes on retinol production and biomass. (C) Effect of *VGB* overexpression on retinol production and biomass. (D) Effect of combinatorial transporter overexpression and ATP enhancement on retinol production and biomass. All values presented are the means of three biological replicates, and error bars represent standard deviations. Statistical analysis was performed using Student's t-test, ∗*P* ​< ​0.05, ∗∗*P* ​< ​0.01, ∗∗∗*P<* 0.001.

Combinatorial overexpression of *PDR3*, *PDR10* or *SNQ2* with *FZO1* led to a decrease in retinol titer, while strains with combinatorial overexpression of *MGM1* showed an increase in retinol titer (Fig. 3B). The most pronounced titer increase was observed in the strain co-overexpressing *PDR10* and *MGM1*, with a total retinol titer that was 33.3 ​% higher compared to the control strain, from 401.26 ​mg/L to 534.82 ​mg/L. Meanwhile, the extracellular titer increased from 342.92 ​mg/L to 494.94 ​mg/L. The intracellular proportions of retinol in strains co-overexpressing *FZO1* with *PDR3*, *PDR10*, and *SNQ2* were 4.6 ​%, 6.1 ​%, and 11.0 ​%, respectively, all lower than the corresponding values when individually overexpressing PDR family proteins. However, the growth of strains overexpressing *FZO1* was adversely affected to varying extents (Fig. 3B), which might be the reason why the retinol titer decreased while its secretion was promoted. Mgm1p is present in a complex with Ugo1p and Fzo1p. Thus, individual overexpression of *FZO1* might excessively promote outer membrane fusion and actually damage the function of healthy mitochondria [[57], [58], [59]].

The electron transport chain facilitates the transfer of electrons from NADH to O_2_ through a redox reaction while simultaneously releasing protons (H^+^) to drive ATP synthesis from ADP. Optimizing the supply of electron donors and acceptors in the electron transport chain has proven more effective than directly manipulating the expression of components within the electron transport chain and ATP synthase [60]. Since O_2_ serves as the most common terminal electron acceptor, improving O_2_ delivery may enhance biosynthesis during aerobic fermentation while strengthening ATP supply. In retinoid production, oxidative cleavage of β-carotene catalyzed by Blh is a crucial step that also requires sufficient O_2_ supply. Vgb is a *Vitreoscilla* hemoglobin that enhances O_2_ transport and boosts energy metabolism [61]. To investigate whether enhanced oxygen supply could strengthen the transport efficiency of endogenous PDR proteins by enhancing ATP synthesis, and concurrently promote retinol production by improving β-carotene cleavage, *VGB* was overexpressed in Y03-43. The overexpression of *VGB* resulted in a 14.1 ​% increase in total retinol titers, from 327.01 ​mg/L to 406.62 ​mg/L (Fig. 3C), albeit with an adverse impact on strain growth. Simultaneously, the extracellular proportion of retinol increased from 85.6 ​% to 88.9 ​%. Vgb may have directly enhanced Blh function by increasing oxygen supply or indirectly strengthened the role of endogenous PDR proteins by enhancing oxidative phosphorylation. The negative effect of *VGB* overexpression on strain growth is likely attributed to heightened O_2_ supply leading to increased generation of singlet oxygen and inducing oxidative stress within the strain [35].

**Fig. 4:**
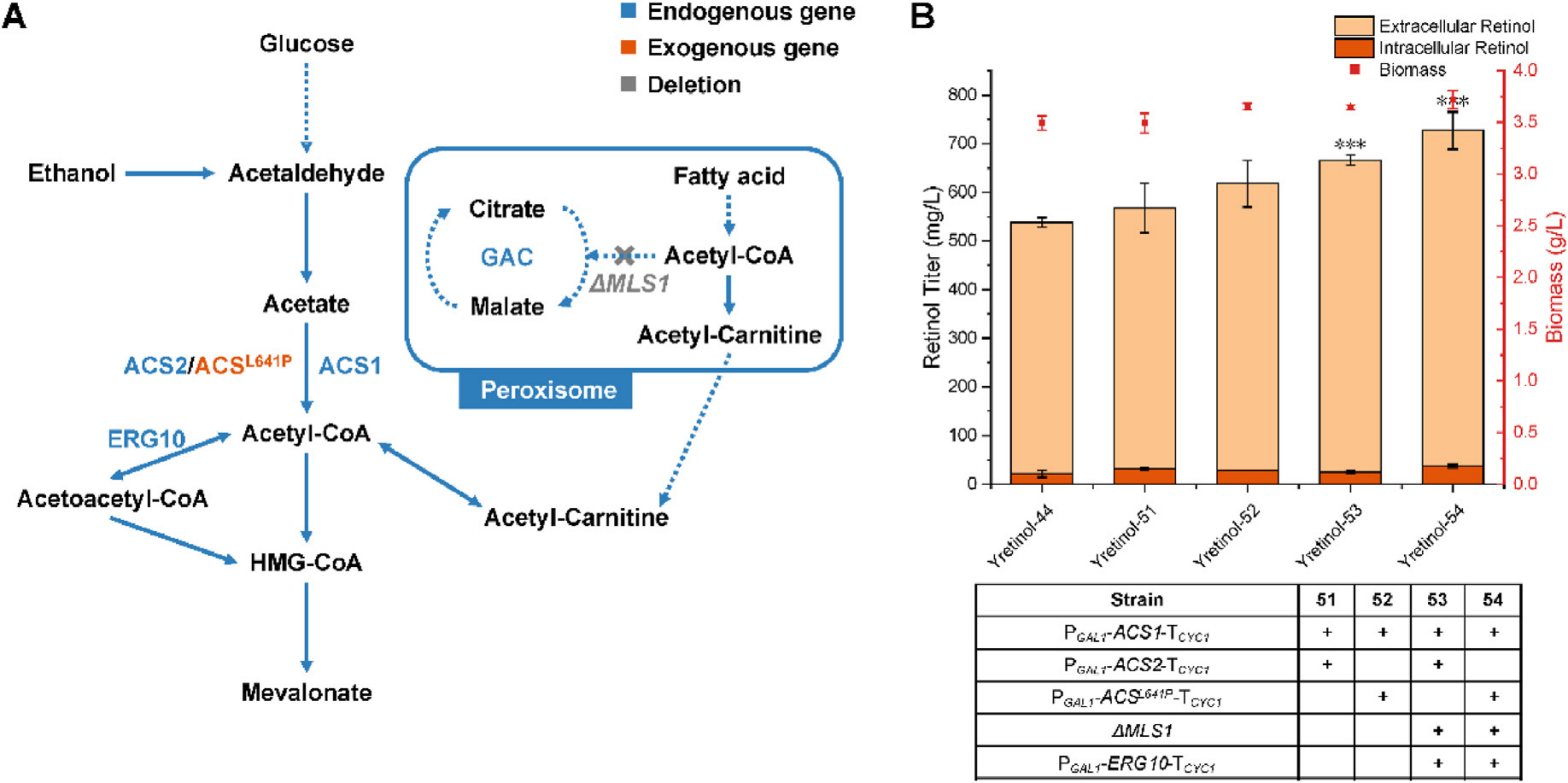
Effect of precursor supply engineering on retinol production. (A) Strategies for strengthening precursor supply. (B) Effect of precursor enhancement on retinol production and biomass. GAC, glyoxylate cycle. All values presented are the means of three biological replicates, and error bars represent standard deviations. Statistical analysis was performed using Student's t-test, ∗*P* ​< ​0.05, ∗∗*P* ​< ​0.01, ∗∗∗*P<* 0.001.

To further enhance retinol transport efficiency, *MGM1* and *VGB* were overexpressed in Yretinol-21 where Pdr3p and Pdr10p were co-overexpressed to achieve maximum retinol production. However, neither individual overexpression of *MGM1* or *VGB* nor co-overexpression of both further increased retinol titers (Fig. 3D). This may be due to insufficient precursor supply within the cells (Fig. S3). To enhance precursor supply further, endogenous *ACS1* and *ACS2* were overexpressed to promote acetyl-CoA synthesis, and the effects of endogenous *ACS2* and *ACS*^*L641P*^ on retinol synthesis were compared. *ACS*^*L641P*^ is an ACS mutant from *Salmonella enterica* that has been reported to exhibit higher acetyl-CoA synthetase activity [62,63]. Meanwhile, the malate synthase gene *MLS1* was knocked out to reduce acetyl-CoA consumption by the glyoxylate cycle [17], and *ERG10* was overexpressed to channel acetyl-CoA into the mevalonate pathway. As expected, retinol titers for Yretinol-53 and Yretinol-54 both showed significant increases (by 23.73 ​% and 35.05 ​%, respectively) (Fig. 4B). Additionally, the combination of *ACS1* and *ACS*^*L641P*^ proved superior to *ACS1* combined with *ACS2*. The final strain Yretinol-54 achieved a retinol titer of 727.30 ​mg/L, 35.0 ​% higher than that of the control strain Yretinol-44.

## 4 Conclusions

**Fig. 5:**
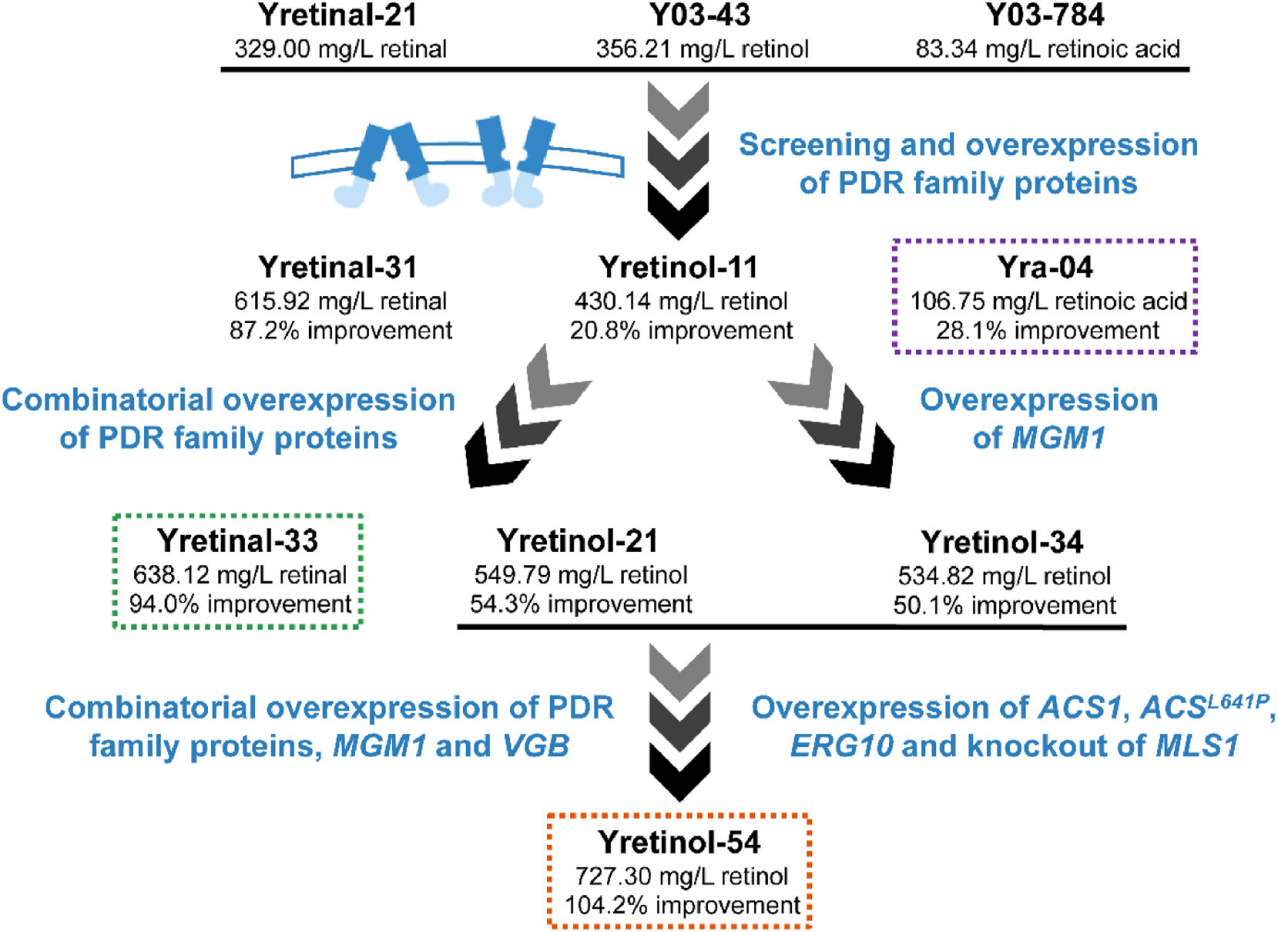
Diagram summarizing the retinoids titer improvement obtained through transporter engineering, ATP and precursor supply enhancement.

Through individual overexpression in yeast strains producing a mixture of retinoids, we screened PDR family proteins that promote the efflux of retinal and retinoic acid. Three proteins (Pdr3p, Pdr10p, Snq2p) were found to promote retinal efflux, while eight proteins (Pdr3p, Pdr8p, Pdr11p, Pdr12p, Pdr18p, Aus1p, Snq2p, Yor1p) were found to enhance retinoic acid production. By engineering transporter overexpression and ATP supply, we obtained a strain Yretinal-33, which selectively produced 638.12 ​mg/L of retinal (with an extracellular proportion of 98.7 ​%), and another strain, Yra-04, which produced 106.75 ​mg/L of retinoic acid (Fig. 5); both represent the highest yields reported in shake-flask cultures. Additionally, Pdr3p, Pdr10p, and Snq2p were shown to promote retinol efflux in engineered strains selectively producing retinol. After combinatorially overexpressing these proteins, enhancing energy metabolism and oxygen supply, and strengthening precursor supply, 727.30 ​mg/L of retinol was produced from 20 ​g/L glucose in shake flasks. The carbon conversion rate of 7.62 ​% exceeds that of the previously reported best producer, which generated 1.21 ​g/L retinol from a mixed carbon source composed of 25 ​g/L glucose, 25 ​g/L sucrose, and 25 ​g/L glycerol [17]. This study demonstrates that transporter engineering combined with ATP and oxygen supply engineering is an efficient strategy for promoting vitamin A biosynthesis. Moreover, enhanced oxygen utilization is expected to reduce the ventilation requirements during large-scale fermentation, and provide a more economical route for vitamin A production. However, the adverse effects of transporter and *VGB* overexpression on the biomass observed in shake-flask cultures suggest the necessity to balance growth and production in high-density fermentation, with dynamic regulation of their expression levels and membrane engineering for improved fluidity and integrity of the cell membranes as possible future directions.

## CRediT authorship contribution statement

**Yijun Zhang:** Investigation, Data curation, Writing – original draft. **Qiongyue Hu:** Methodology. **Hongwei Yu:** Conceptualization, Project administration. **Lidan Ye:** Conceptualization, Supervision, Writing – review & editing, Funding acquisition.

## Data availability

Data will be made available on request.

## Declaration of competing interest

The authors declare that they have no known competing financial interests or personal relationships that could have appeared to influence the work reported in this paper.
