# Supplementary material for: Enhanced vitamin A production by engineering transporters, ATP and precursor supply
Source: Biodes Res. 2025 Apr 17;7(2):100023. doi: 10.1016/j.bidere.2025.100023 (PMC12710018; doi:10.1016/j.bidere.2025.100023)
Supplement: Multimedia component 1 [file mmc1.docx]

**Supplementary Information**

**Enhanced vitamin A production by engineering transporters, ATP and precursor supply**

Yijun Zhang ^a,b^, Qiongyue Hu ^b^, Hongwei Yu ^a,b*^, Lidan Ye ^a, b*^

^a^ *Key Laboratory of Biomass Chemical Engineering (Education Ministry), College of Chemical and Biological Engineering, Zhejiang University, Hangzhou 310058, China*

^b^ *Institute of Bioengineering, College of Chemical and Biological Engineering, Zhejiang University, Hangzhou 310058, China*


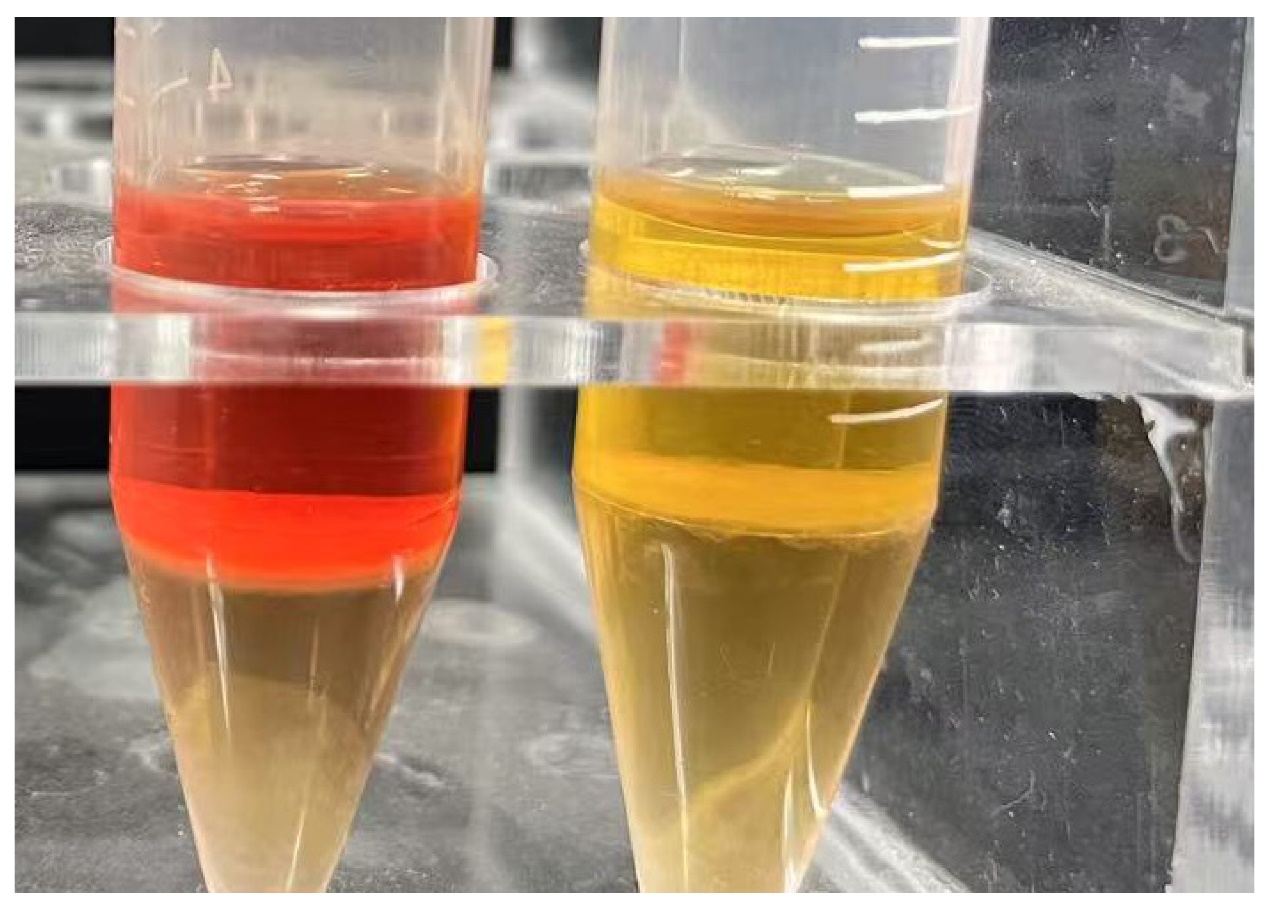


**Fig. S1. Color of the dodecane phase in strains with (left) or without (right) transporter overexpression**

**
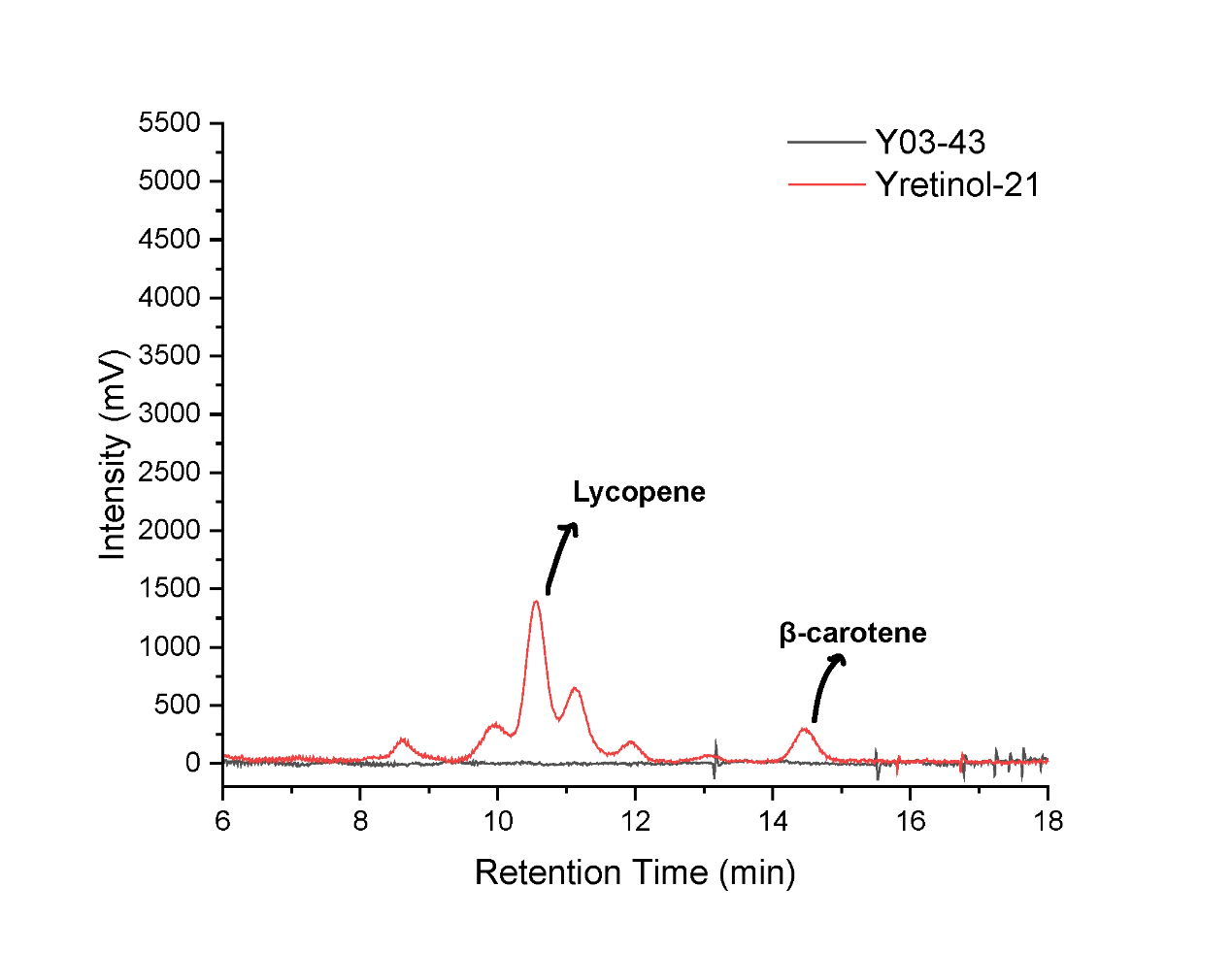
**

**Fig. S2. HPLC analysis of precursors in the organic phase for strains Y03-43 and Yretinol-21**


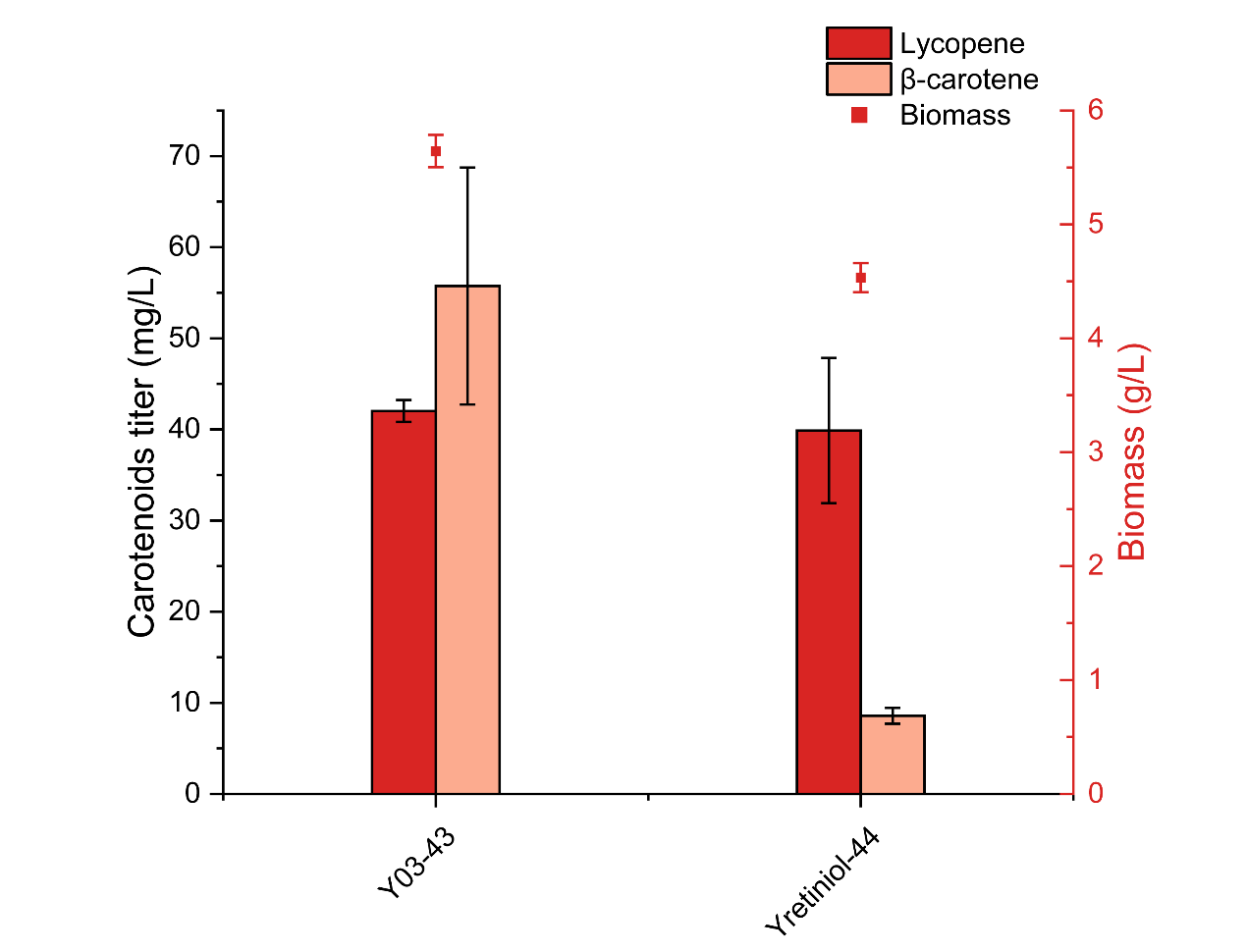


**Fig. S3. Carotenoid production in strains before (Y03-43) and after (Yretinol-44) transporter engineering**

**Table S1** Plasmids used in this study

| **Plasmid name** | **Genotype/ Description** | **Reference** |
| --- | --- | --- |
| pUMRI-ROX1 | *loxp-KanMX-URA3-pbr322ori-loxp,*  T*_ADH1_*-*MCS1*-P*_GAL10_*-P*_GAL1_*-*MCS2*-T*_CYC1_*,  *ROX1* homologous arm | ^1^ |
| pUMRI-911B | *loxp-KanMX-URA3-pbr322ori-loxp,*  T*_ADH1_*-*MCS1*-P*_GAL10_*-P*_GAL1_*-*MCS2*-T*_CYC1_*,  *911B* homologous arm | This study |
| pUMRI-ROX1-*PDR1* | *loxp-KanMX-URA3-pbr322ori-loxp,*  T*_ADH1_*-*MCS1*-P*_GAL10_*-P*_GAL1_*-*PDR1*-T*_CYC1_*,  *ROX1* homologous arm | This study |
| pUMRI-ROX1-*PDR3* | *loxp-KanMX-URA3-pbr322ori-loxp,*  T*_ADH1_*-*MCS1*-P*_GAL10_*-P*_GAL1_*-*PDR3*-T*_CYC1_*,  *ROX1* homologous arm | This study |
| pUMRI-ROX1-*PDR5* | *loxp-KanMX-URA3-pbr322ori-loxp,*  T*_ADH1_*-*MCS1*-P*_GAL10_*-P*_GAL1_*-*PDR5*-T*_CYC1_*,  *ROX1* homologous arm | This study |
| pUMRI-ROX1-*PDR8* | *loxp-KanMX-URA3-pbr322ori-loxp,*  T*_ADH1_*-*MCS1*-P*_GAL10_*-P*_GAL1_*-*PDR8*-T*_CYC1_*,  *ROX1* homologous arm | This study |
| pUMRI-ROX1-*PDR10* | *loxp-KanMX-URA3-pbr322ori-loxp,*  T*_ADH1_*-*MCS1*-P*_GAL10_*-P*_GAL1_*-*PDR10*-T*_CYC1_*,  *ROX1* homologous arm | This study |
| pUMRI-ROX1-*PDR11* | *loxp-KanMX-URA3-pbr322ori-loxp,*  T*_ADH1_*-*MCS1*-P*_GAL10_*-P*_GAL1_*-*PDR11*-T*_CYC1_*,  *ROX1* homologous arm | This study |
| pUMRI-ROX1-*PDR12* | *loxp-KanMX-URA3-pbr322ori-loxp,*  T*_ADH1_*-*MCS1*-P*_GAL10_*-P*_GAL1_*-*PDR12*-T*_CYC1_*,  *ROX1* homologous arm | This study |
| pUMRI-ROX1-*PDR15* | *loxp-KanMX-URA3-pbr322ori-loxp,*  T*_ADH1_*-*MCS1*-P*_GAL10_*-P*_GAL1_*-*PDR15*-T*_CYC1_*,  *ROX1* homologous arm | This study |
| pUMRI-ROX1-*PDR18* | *loxp-KanMX-URA3-pbr322ori-loxp,*  T*_ADH1_*-*MCS1*-P*_GAL10_*-P*_GAL1_*-*PDR18*-T*_CYC1_*,  *ROX1* homologous arm | This study |
| pUMRI-ROX1-*AUS1* | *loxp-KanMX-URA3-pbr322ori-loxp,*  T*_ADH1_*-*MCS1*-P*_GAL10_*-P*_GAL1_*-*AUS1*-T*_CYC1_*,  *ROX1* homologous arm | This study |
| pUMRI-ROX1-*SNQ2* | *loxp-KanMX-URA3-pbr322ori-loxp,*  T*_ADH1_*-*MCS1*-P*_GAL10_*-P*_GAL1_*-*SNQ2*-T*_CYC1_*,  *ROX1* homologous arm | This study |
| pUMRI-ROX1-*YOR1* | *loxp-KanMX-URA3-pbr322ori-loxp,*  T*_ADH1_*-*MCS1*-P*_GAL10_*-P*_GAL1_*-*YOR1*-T*_CYC1_*,  *ROX1* homologous arm | This study |
| pUMRI-ROX1-*YOL075C* | *loxp-KanMX-URA3-pbr322ori-loxp,*  T*_ADH1_*-*MCS1*-P*_GAL10_*-P*_GAL1_*-*YOL075C*-T*_CYC1_*,  *ROX1* homologous arm | This study |
| pUMRI-ROX1-*PDR3*-*FZO1* | *loxp-KanMX-URA3-pbr322ori-loxp,*  T*_ADH1_*-*FZO1*-P*_GAL10_*-P*_GAL1_*-*PDR3*-T*_CYC1_*,  *ROX1* homologous arm | This study |
| pUMRI-ROX1-*PDR3*-*MGM1* | *loxp-KanMX-URA3-pbr322ori-loxp,*  T*_ADH1_*-*MGM1*-P*_GAL10_*-P*_GAL1_*-*PDR3*-T*_CYC1_*,  *ROX1* homologous arm | This study |
| pUMRI-ROX1-*PDR10*-*FZO1* | *loxp-KanMX-URA3-pbr322ori-loxp,*  T*_ADH1_*-*FZO1*-P*_GAL10_*-P*_GAL1_*-*PDR10*-T*_CYC1_*,  *ROX1* homologous arm | This study |
| pUMRI-ROX1-*PDR10*-*MGM1* | *loxp-KanMX-URA3-pbr322ori-loxp,*  T*_ADH1_*-*MGM1*-P*_GAL10_*-P*_GAL1_*-*PDR10*-T*_CYC1_*,  *ROX1* homologous arm | This study |
| pUMRI-ROX1-*SNQ2*-*FZO1* | *loxp-KanMX-URA3-pbr322ori-loxp,*  T*_ADH1_*-*FZO1*-P*_GAL10_*-P*_GAL1_*-*SNQ2*-T*_CYC1_*,  *ROX1* homologous arm | This study |
| pUMRI-ROX1-*SNQ2*-*MGM1* | *loxp-KanMX-URA3-pbr322ori-loxp,*  T*_ADH1_*-*MGM1*-P*_GAL10_*-P*_GAL1_*-*SNQ2*-T*_CYC1_*,  *ROX1* homologous arm | This study |
| pUMRI-ROX1-*VGB* | *loxp-KanMX-URA3-pbr322ori-loxp,*  T*_ADH1_*- *MCS1*-P*_GAL10_*-P*_GAL1_*-*VGB*-T*_CYC1_*,  *ROX1* homologous arm | This study |
| pUMRI-ROX1-*PDR3*-*PDR10* | *loxp-KanMX-URA3-pbr322ori-loxp,*  T*_ADH1_*-*PDR10*-P*_GAL10_*-P*_GAL1_*-*PDR3*-T*_CYC1_*,  *ROX1* homologous arm | This study |
| pUMRI-ROX1-*PDR3*-*SNQ2* | *loxp-KanMX-URA3-pbr322ori-loxp,*  T*_ADH1_*-*SNQ2*-P*_GAL10_*-P*_GAL1_*-*PDR3*-T*_CYC1_*,  *ROX1* homologous arm | This study |
| pUMRI-ROX1-*PDR10*-*PDR3* | *loxp-KanMX-URA3-pbr322ori-loxp,*  T*_ADH1_*-*PDR3*-P*_GAL10_*-P*_GAL1_*-*PDR10*-T*_CYC1_*,  *ROX1* homologous arm | This study |
| pUMRI-ROX1-*PDR10*-*SNQ2* | *loxp-KanMX-URA3-pbr322ori-loxp,*  T*_ADH1_*-*SNQ2*-P*_GAL10_*-P*_GAL1_*-*PDR10*-T*_CYC1_*,  *ROX1* homologous arm | This study |
| pUMRI-ROX1-*SNQ2*-*PDR3* | *loxp-KanMX-URA3-pbr322ori-loxp,*  T*_ADH1_*-*PDR3*-P*_GAL10_*-P*_GAL1_*-*SNQ2*-T*_CYC1_*,  *ROX1* homologous arm | This study |
| pUMRI-ROX1-*SNQ2*-*PDR10* | *loxp-KanMX-URA3-pbr322ori-loxp,*  T*_ADH1_*-*PDR10*-P*_GAL10_*-P*_GAL1_*-*SNQ2*-T*_CYC1_*,  *ROX1* homologous arm | This study |
| pUMRI-911B-*VGB* | *loxp-KanMX-URA3-pbr322ori-loxp,*  T*_ADH1_*-*MCS1*-P*_GAL10_*-P*_GAL1_*-*VGB*-T*_CYC1_*,  *911B* homologous arm | This study |
| pUMRI-911B-*MGM1* | *loxp-KanMX-URA3-pbr322ori-loxp,*  T*_ADH1_*-*MGM1*-P*_GAL10_*-P*_GAL1_*-*MCS2*-T*_CYC1_*,  *911B* homologous arm | This study |
| pUMRI-911B-*VGB*-*MGM1* | *loxp-KanMX-URA3-pbr322ori-loxp,*  T*_ADH1_*-*MGM1*-P*_GAL10_*-P*_GAL1_*-*VGB*-T*_CYC1_*,  *911B* homologous arm | This study |
| pUMRI-911B-*ACS1* | *loxp-KanMX-URA3-pbr322ori-loxp,*  T*_ADH1_*- *MCS1*-P*_GAL10_*-P*_GAL1_*-A*CS1*-T*_CYC1_*,  *911B* homologous arm | This study |
| pUMRI-911B-*ACS2* | *loxp-KanMX-URA3-pbr322ori-loxp,*  T*_ADH1_*- *MCS1*-P*_GAL10_*-P*_GAL1_*-*ACS2*-T*_CYC1_*,  *911B* homologous arm | This study |
| pUMRI-911B-*ACS^L641P^* | *loxp-KanMX-URA3-pbr322ori-loxp,*  T*_ADH1_*- *MCS1*-P*_GAL10_*-P*_GAL1_*-*ACS^L641P^* -T*_CYC1_*,  *911B* homologous arm | This study |
| pUMRI-911B-*ERG10* | *loxp-KanMX-URA3-pbr322ori-loxp,*  T*_ADH1_*- *MCS1*-P*_GAL10_*-P*_GAL1_*-*ERG10*-T*_CYC1_*,  *911B* homologous arm | This study |
| P416-Cas9-G418 | CEN/ARS, P*_TEF1_*-*Cas9*-T*_CYC1_*, P*_TEF1_*-*Kan*-T*_TEF1_* | ^2^ |
| P426-SpSgH | *2μori*-*Amp*-*pbr322ori*, URA3,  P*_SNR52_*-*gRNA scaffold*-T*_SUP4_* | ^3^ |
| P426-ccdB | *2μori*-*Amp*-*pbr322ori*, URA3, ccdB | ^3^ |
| P426-SgInt7-18 | *2μori*-*Amp*-*pbr322ori*, URA3,  P*_SNR52_*-*Int7*-T*_SUP4_*-P*_SNR52_*-*Int18*-T*_SUP4_* | ^3^ |
| P426-SpSgH-*ADH6* | *2μori*-*Amp*-*pbr322ori*, URA3,  P*_SNR52_*-*ADH6*-T*_SUP4_* | This study |
| P426-SpSgH-*ADH7* | *2μori*-*Amp*-*pbr322ori*, URA3,  P*_SNR52_*-*ADH7*-T*_SUP4_* | This study |
| P426-SpSgH-*GRE2* | *2μori*-*Amp*-*pbr322ori*, URA3,  P*_SNR52_*-*GRE2*-T*_SUP4_* | This study |
| P426-SpSgH-*SFA1* | *2μori*-*Amp*-*pbr322ori*, URA3,  P*_SNR52_*-*SFA1*-T*_SUP4_* | This study |
| P426-SpSgH-*ENV9* | *2μori*-*Amp*-*pbr322ori*, URA3,  P*_SNR52_*-*ENV9*-T*_SUP4_* | This study |
| P426-SpSgH-*MLS1* | *2μori*-*Amp*-*pbr322ori*, URA3,  P*_SNR52_*-*MLS1*-T*_SUP4_* | This study |
| P426-ccdB-*ADH6*-*ADH7* | *2μori*-*Amp*-*pbr322ori*, URA3,  P*_SNR52_*-*ADH6*-T*_SUP4_*-P*_SNR52_*-*ADH7*-T*_SUP4_* | This study |
| P426-ccdB-*GRE2*-*SFA1* | *2μori*-*Amp*-*pbr322ori*, URA3,  P*_SNR52_*-*GRE2*-T*_SUP4_*-P*_SNR52_*-*SFA1*-T*_SUP4_* | This study |

**Table S2** Primers used in this study

| **Primer name** | **Sequence (5’-3’)** | **Description** |
| --- | --- | --- |
| PDR1-BamHⅠ-F | CGCGGATCCATGCGAGGCTTGACACCTAAGAACGGTGTACATA | For amplification of genes |
| PDR1-KpnⅠ-R | CGGGGTACCTTAACTATCTGGATAAACGTCGCTCCACAGGATAC |  |
| PDR3-BamHⅠ-F | CGCGGATCCATGAAAGTGAAGAAATCAACTAGATCAAAAGTT |  |
| PDR3-KpnⅠ-R | CGGGGTACCTCATAAGAAGGGATATGAAGTATTGTCATTCC |  |
| PDR5-XmaⅠ-F | CCCCCCGGGATGCCCGAGGCCAAGCTTAACAATAACGTCAACG |  |
| PDR5-XhoⅠ-R | CCGCTCGAGTTATTTCTTGGAGAGTTTACCGTTCTTTTTAGGCA |  |
| PDR8-XmaⅠ-F | CCCCCCGGGATGGATGGATCCCATTTTCCTATGAAATCTACTACCGGAGAGCCCGTTT |  |
| PDR8-KpnⅠ-R | CGGGGTACCTTATAAATCGAAATGATATTGTTTATAAAATTTCTTTTTGTCAATGGAC |  |
| PDR10-XmaⅠ-F | CCCCCCGGGATGTTGCAAGCGCCCTCAAGTTCAAACTCGGGTT |  |
| PDR10-KpnⅠ-R | CGGGGTACCTTATTTCTTTAATTTTTTGCTTTTCTTTGGAACCCG |  |
| PDR11-BamHⅠ-F | CGCGGATCCATGTCTCTTTCCAAATATTTTAATCCAATTCCTGACGCTTCAGTCACCT |  |
| PDR11-XmaⅠ-R | CCCCCCGGGTTATACGCTTTGTTCGTTTGGATTATGAGATGTTTTGGACCGCCTCATT |  |
| PDR12-XmaⅠ-F | CCCCCCGGGATGTCTTCGACTGACGAACATATTGAGAAAGACATTTCGTCGAGATCGA |  |
| PDR12-XhoⅠ-R | CCGCTCGAGTTATTTCTTCGTGATTTTATTTTCGTCACCTGGAACTGTTTGGAAAATG |  |
| PDR15-BamH1-F | CGCGGATCCATGTCATCAGATATCAGAGACGTAGAG |  |
| PDR15-Sal1-R | ACGGTCGACTCACTTCTTGGGTTTTTCGGAAA |  |
| PDR18-BamH1-F | CGCGGATCCATGGAATGCGTTTCAGTAGAAG |  |
| PDR18-Kpn1-R | CGGGGTACCCTAAATGAAACCGAAGTTTCTCCATAAG |  |
| AUS1-XmaⅠ-F | CCCCCCGGGATGTCAATTTCAAAGTACTTCACTCCCGTTGCTGACGGGTCACTCACTTT |  |
| AUS1-XhoⅠ-R | CCGCTCGAGTTAGTTCTGTACAGGCTTCTTCCCTCTGTGTGGAATTACTTTGGTGATA |  |
| YOR1-XmaⅠ-F | CCCCCCGGGATGACGATTACCGTGGGGGATGCAGTTTCGGAGA |  |
| YOR1-XhoⅠ-R | CCGCTCGAGTTAACTTCTGTTCTCGAAATCATTTTCCACAATA |  |
| SNQ2-BamHⅠ-F | CGCGGATCCATGAGCAATATCAAAAGCACGCAAGATAGCTCTC |  |
| SNQ2-XmaⅠ-R | CCCCCCGGGTTACTGCTTCTTTTTCCTTATGTTTTTAATTTTA |  |
| YOL075C-BamHⅠ-F | CGCGGATCCATGTCACAGCAGGAGAATGGTGATGTGGCCACTG |  |
| YOL075C-KpnⅠ-R | CGGGGTACCTCACCATTTTATCCACTCCAATTTTGCTTTTAAA |  |
| FZO1-EcoRⅠ-F | CCGGAATTCATGTCTGAAGGAAAACAACAATTCAAAGACAGCAATAAACCGCACAAGGA |  |
| FZO1-NotⅠ-R | AAGGAAAAAAGCGGCCGCCTAATCGATGTCTAAATTTATTTCTTCCACCATCAATTTTT |  |
| MGM1-NotⅠ-F | AAGGAAAAAAGCGGCCGCATGAATGCGAGCCCAGTACGGCTTTTAATTCTGAGAAGACA |  |
| MGM1-SacⅠ-R | CGAGCTCTCATAAATTTTTGGAGACGCCCTTGTAGCTTTTCTTGAAAACCAAGATTGAA |  |
| VGB-SalⅠ-F | ACGCGTCGACATGTTGGATCAACAAACTATTAATATTATTAAGGCTACTGTT |  |
| VGB-SacⅡ-R | TCCCCGCGGTTATTCAACAGCTTGAGC |  |
| ACS1-SalⅠ-F | ACGCGTCGACATGTCGCCCTCTGCC |  |
| ACS1-KpnⅠ-R | CGGGGTACCTTACAACTTGACCGAATCAATTAGATGTCTAACAATG |  |
| ACS2-BamHⅠ-F | CGCGGATCCTTATTTCTTTTTTTGAGAGAAAAATTGGTTCTCTACAGC |  |
| ACS2-SacⅡ-R | TCCCCGCGGATGACAATCAAGGAACATAAAGTAG |  |
| ACS2*^L641P^*-SalⅠ-F | ACGCGTCGACTCATGATGGCATAGCAATAGCTTG |  |
| ACS2*^L641P^*-KpnⅠ-R | CGGGGTACCATGTCACAAACACACAAACATGCTATT |  |
| ERG10-BamHI-F | CGCGGATCCATGTCTCAGAACGTTTACATTGTATCG |  |
| ERG10-XhoI-R | CCGCTCGAGTCATATCTTTTCAATGACAATAGAGGAAGC |  |
| GAL1F | TCTGGGGTAATTAATCAGCG | For verifying plasmids construction and gene integration |
| GAL10F | AACTTCTTTGCGTCCATCCA |  |
| rox1HA-UP-R | ACCCAGGTGCAGGCGTACTTTAAAGTATTT |  |
| rox1HA-DOWN-R | TGTGTCTATATCTACAATATGAACCTGTTTAACAAAGCTTAACATTGTC |  |
| 911BHA-UP-R | GATGAGATATGGAGGTGTTTTTACGTTTTTGAGTGCTATAACTT |  |
| 911BHA-DOWN-R | CCATCCTTCATTAGCCCGGCTCAA |  |
| SpSgH-ADH6-F | GATCGACGGCTATGTGTCGCAGGG | CRISPR Guide |
| SpSgH-ADH6-R | AAACCCCTGCGACACATAGCCGTC |  |
| SpSgH-ADH7-F | GATCCTAGCCGCTCCATTATTGTG |  |
| SpSgH-ADH7-R | AAACCACAATAATGGAGCGGCTAG |  |
| SpSgH-GRE2-F | GATCGACCCAGTTAACGCCTACTG |  |
| SpSgH-GRE2-R | AAACCAGTAGGCGTTAACTGGGTC |  |
| SpSgH-SFA1-F | GATCGTAGGACTCTCCGTTATCCA |  |
| SpSgH-SFA1-R | AAACTGGATAACGGAGAGTCCTAC |  |
| SpSgH-ENV9-F | GATCCTGGAAACTGAGCAAGACGT |  |
| SpSgH-ENV9-R | AAACACGTCTTGCTCAGTTTCCAG |  |
| SpSgH-MLS1-F | GATCAGATTACATTGGGATCCCAA |  |
| SpSgH-MLS1-R | AAACTTGGGATCCCAATGTAATCT |  |
| URA3-Ver/seq-F | GGTAGAGGGTGAACGTTACAG |  |
| GRNA-Ver/Seq-R | TGTGGAATTGTGAGCGGATAAC |  |
| Donor-ADH6-F | GACTTTCACATCCACATTCGAGGAAGAAATTCAACACAACAACAAGAAAAGCCAAAATC | For amplification of donors |
| Donor-ADH6-R | TTAAAAAGAAAGGAGCTACATTTATCAAGAGCTTGACAACGATTTTGGCTTTTCTTGTT |  |
| Donor-ADH7-F | TTGCTTGATATTATTGAAATAAAAAAAAACATAGAACCACTGAAAAATACAAAAAAAAA |  |
| Donor-ADH7-R | ATGCATTTTAAGAGATTCTGAAAAATATTACGTATATAGATTTTTTTTTGTATTTTTCA |  |
| Donor-SFA1-F | GTTAAGTTTACTTCTACAAAATCTCCAAGTAAAGAAGGAATATAAGTAATATAAGTACA |  |
| Donor-SFA1-R | GAAAATTTGAGTCATGCTTACTTAGTTTAATTAAGTACTCTGTACTTATATTACTTATA |  |
| Donor-GRE2-F | GTTGATATAACGTGTACGATTTTCAAACAAACAGATAGCAGTATCACACGCCCGTAAAT |  |
| Donor-GRE2-R | ACGTTAATATATATAAATATTATCTATTTTCATTTAAAGTATTTACGGGCGTGTGATAC |  |
| Donor-ENV9-F | TTAGAGGCATCCGCAGTACGAGACATACGCCAAAGGCAGACAAGACGGAAAGATTGAAA |  |
| Donor-ENV9-R | AAAATGTACATCTATCTACTCTTTCAATCTTTCCGTCTTGTCTGCCTTTGGCGTATGTC |  |
| Donor-MGM1-F | ATTATTGAAATAAAAAAAAACATAGAACCACTGAAAAATACAAAAAAAAAATGAATGCGAGCCCAGTACGGCTTTTAAT |  |
| Donor-MGM1-R | GAGATTTGACATGCATTTTAAGAGATTCTGAAAAATATTACGTATATAGATCATAAATTTTTGGAGACGCCCTTGTAGC |  |
| Donor-VGB-F | AACAAGAAAAGCCAAAATCTGTGCCTCGCGCCGCACTGCTCCGAACAATAAAGATTCTA |  |
| Donor-VGB-R | TTATCAAGAGCTTGACAACCTTCGAGCGTCCCAAAACCTTCTCAAGCAAGGTTTTCAGT |  |
| Int7-Donor-F | CGGACTATTGCTGTCTTCTCGTGGTAAATGCGTGTTCCAGCTTCGAGCGTCCCAAAACC |  |
| Int7-Donor-R | AACTTAACAAATCGGCAACACTTTTATGGGGCCCCGCTCGTGTGCCTCGCGCCGCACTG |  |
| Int18-Donor-F | AATGGCACTGAGAGACGTTTTTGCCAAACAAGTACTAAATCTTCGAGCGTCCCAAAACC |  |
| Int18-Donor-R | CTTATAGCATACTATTTTAGTTATGAATTGTGAAAATCCTTGTGCCTCGCGCCGCACTG |  |
| Donor-MLS1-F | ACTAAACAAAGTAGTAAAAGCACATAAAAGAATTAAGAAATGTGCCTCGCGCCGCACTG |  |
| Donor-MLS1-R | GAATATATTTTTATATATGTGTACACTGGGGCAAGGGAGACTTCGAGCGTCCCAAAACC |  |
| 4gRNA-F1 | NNNNNGGTCTCCGGACTCTTTGAAAAGATAATGTATG | For multi-guide plasmid construction |
| 4gRNA-R1 | NNNNNGGTCTCCCGGACTTGCATGCCTGCAGGGAGCTC |  |
| 4gRNA-F2 | NNNNNGGTCTCCTCCGTCTTTGAAAAGATAATGTATG |  |
| 4gRNA-R2 | NNNNNGGTCTCCCTGGCTTGCATGCCTGCAGGGAGCTC |  |
| 4gRNA-F3 | NNNNNGGTCTCCCCAGTCTTTGAAAAGATAATGTATG |  |
| 4gRNA-R3 | NNNNNGGTCTCCGCTGCTTGCATGCCTGCAGGGAGCTC |  |
| 4gRNA-F4 | NNNNNGGTCTCCCAGCTCTTTGAAAAGATAATGTATG |  |
| 4gRNA-R4 | NNNNNGGTCTCCCAACCTTGCATGCCTGCAGGGAGCTC |  |
| ADH6-UP-F | CAACTTGGTAATGACGTACTGGATACTTTCGAC | For verifying gene integration |
| ADH6-DOWN-R | GATCGGGAGGGGTGAAACTACCCTTGA |  |
| ADH7-UP-F | TAGATCAGGGACTATGCGAGCGACAAG |  |
| ADH7-DOWN-R | GGACCTCATCAGACATGCTCGGACT |  |
| GRE2-UP-F | TTACCGCGTGAACTATGTCATATTTGCGAT |  |
| GRE2-DOWN-R | AAATATCTACTTCTTGTGGGGAGACGGGTAGA |  |
| SFA1-UP-F | TCCTTTGCATCAGGTCAATCGTGACAG |  |
| SFA1-DOWN-R | CAGCGTCGCGTACTCCCAGTAATAACAATATTT |  |
| ENV9-UP-F | ACACTCACTCTCGTCAGAAAAATCTGTCGAG |  |
| ENV9-DOWN-R | GGCTGTAGGGCTTCCTTCTTCCTTCTT |  |
| Int7-VER-F | CGCCAAGGTTTACTGACAGAGAC |  |
| Int7-VER-R | ACGAGGGCATTACGATGGCTTC |  |
| Int18-VER-F | ACAAGCGCCCATTCTGACCATTAAACT |  |
| Int18-VER-R | AAATGCAAGTGTATCGACATTTTTGAATGATCATGTTGAG |  |
| MLS1-UP-F | ATATCATAGCTTCCATTGGGCCGATGAAGTTAG |  |
| MLS1-DOWN-R | GGGTATTAGCAGGGATAGGCGTGTACATTTC |  |

**Supplementary references**

(1) Hu, Q.; Zhang, T.; Yu, H.; Ye, L. Selective biosynthesis of retinol in *S. cerevisiae*. *Bioresources and Bioprocessing* **2022**, *9* (1), 22. DOI: 10.1186/s40643-022-00512-8.

(2) Li, M.; Zhou, P.; Chen, M.; Yu, H.; Ye, L. Spatiotemporal Regulation of Astaxanthin Synthesis in *S. cerevisiae*. *ACS Synthetic Biology* **2022**, *11* (8), 2636-2649. DOI: 10.1021/acssynbio.2c00044.

(3) Lian, J.; HamediRad, M.; Hu, S.; Zhao, H. Combinatorial metabolic engineering using an orthogonal tri-functional CRISPR system. *Nature Communications* **2017**, *8* (1), 1688. DOI: 10.1038/s41467-017-01695-x.
